# Does hair dye use really increase the risk of prostate cancer?

**DOI:** 10.1186/s12885-017-3656-z

**Published:** 2017-11-07

**Authors:** Bang-Ping Jiann

**Affiliations:** 1 Division of Basic Medical Research, Department of Medical Education and Research, Kaohsiung Veterans General Hospital, 386, Dajhong 1st Road, Zuoying District, Kaohsiung, 813 Taiwan; 2 School of Medicine, National Yang-Ming University, No.155, Sec.2, Linong Street, Taipei, 112 Taiwan

**Keywords:** Hair dyes, Prostate cancer, Personal use, External validity

## Abstract

Recently, Shu-Yu Tai et al. reported that personal hair dye use increased risk of prostate cancer with a dose-response effect. Although hair dyes were identified as carcinogenic in animals and increased risk of some cancers among hairdressers, the existing epidemiological data did not support that personal hair dye use increased risk of cancers, even for bladder cancer. Given that Tai et al.’s report of a potential hazard of personal hair dye use on risk of prostate cancer was particular, the methodology of the study was scrutinized and some flaws were found including the issue of external validity.

## Main text

Aromatic amines in hair dyes were identified as carcinogenic in animals [1]. In the past decades, whether hair dye use increased risk of cancers in humans gained much concern and many observational studies on this field have been published. Although a significant increase of some cancers including hematopoietic and bladder cancers were observed among hairdressers [2], meta-analysis of epidemiologic studies did not show strong evidence of marked increase in risk of cancer among personal hair dye users [3]. The International Agency for Research on Cancer claimed that personal hair dye use was “not classifiable as to its carcinogenicity to humans” [4], and some controversies still need to be clarified.

A recently published case-control study by Tai et al. reported that personal hair dye use increased the risk of prostate cancer with a dose-response effect [5]. The authors explained this finding as “the carcinogens could possibly be absorbed through the urothelial epithelium and accumulate in the prostate gland, contributing to the malignancy of the prostate” [5]. To our best knowledge, no study investigated the association of personal hair due use with the risk of prostate cancer. Instead, studies targeted on hairdressers observed no increased risk of prostate cancer [2]. Compared with the prostate, the urinary bladder is in contact with urine for more extended periods; if the carcinogens contained in urine truly increase prostate cancer, urinary bladder would hardly be spared under the same circumstance. The association of personal hair dye use and bladder cancer has been extensively assessed, but the results of epidemiological studies do not indicate a causal association between them [6]. Tai et al.’s report was the first to show a positive association between personal hair dye use and risk of prostate cancer. Because of this particular finding, the methodology of the study needs to be challenged for the validity of the findings.

The authors conducted a case-control study to compare the habits of hair dye use between 296 cases with newly diagnosed prostate cancer and 296 age-matched controls who received a health check-up during Aug., 2000 and Dec., 2008 in two medical centers located in southern Taiwan [5]. The information of hair dye use was obtained through a structured interview. The prevalence of hair dye use was higher in the cases than the controls (95/296 = 32.1% vs. 64/296 = 21.6%, *P* < 0.05), and the hair dye users had increased odds of prostate cancer when compared with the non-users (adjusted odds ratio (OR), 2.15; 95% confidence interval (CI): 1.32–3.57) [5]. Meanwhile, the prognostic outcome was also evaluated among 608 cases with newly diagnosed prostate cancer between August 2000 and December 2007, of whom 227 cases came from the aforementioned 296 cases and the other 381 cases were recruited from a different medical center located in northern Taiwan. Of the 608 cases, 26.4% (161/608) reported having used hair dyes [5]. Based on the above figures, the estimated prevalence of hair dye use in the 381 cases recruited from a different hospital was 23.1% (88/381), which was close to 21.6% (64/296) in the controls. This finding cast a concern about the external validity of Tai et al.’s findings. Besides, a dose-response relationship is one of the criteria for conclusions about causality. The authors reported a dose-response relationship between hair dye use and risk of prostate cancer in terms of duration and frequency [5], but cumulative exposure dose, a critical indicator to estimate a dose-response relationship, was not assessed in the study.

It was possible that in case-control studies, recall bias could facilitate the memory of past exposure more often in cases than in controls. To the contrary, the cases were more likely to have missing data regarding the history of hair dye use than the controls in Tai et al.’s study. Besides, a peculiar phenomenon existed in their results as that the groups with missing data regarding the habit of hair dye use always had a higher OR of prostate cancer than those with the data available. For example, as compared with non-hair dye users, the odds of prostate cancer was 1.73 times (95% CI: 0.91–3.32) for the group with using-frequency ≤ 6 times/year, 2.65 times (95% CI: 1.26–5.78) for the group with using-frequency > 6 times/year, and 3.85 times (95% CI: 1.41–11.74) for the group with missing using-frequency [5]. There are two probable scenarios to explain why the group with missing using-frequency possessed the highest odds of prostate cancer. One scenario is that those with prostate cancer were easier to forget hair dye use habits than those without. Another is, if a positive relationship did exist between the exposure doses and risk of prostate cancer, the more frequent the use of hair dye, the easier to forget such a habit. However, both scenarios did not make sense.

In the mid- to late 1970s, the manufactures reformulated the hair dye products to eliminate some of the ingredients that were carcinogenic. Tai et al. also tried to prove that hair dyes manufactured before the year of 1980 were carcinogenic by dichotomizing the users according to the year of their first use of hair dyes being before or after 1980. The results did show that those starting using hair dyes before 1980 had a significantly increased odds of prostate cancer, whereas those starting using after 1980 did not [5]. However, in this study, most of the hair dye users started their first use before 1980 (*N* = 131), and only few users began to use hair dyes after 1980 (*N* = 8) [5]. Because the number of the users after 1980 was too small, it was inappropriate to compare the risk before and after the year of 1980.

Another issue was the interpretation of OR. In the results, the authors described the adjusted OR of 2.15 as “*the development of prostate cancer in hair dye users was 2.15-fold higher than that in nonusers”.* Two errors existed in this statement. First, the “2.15-fold higher than” should be corrected as “2.15-fold of” or “115% higher than”. Second, the significance of OR indicated an odds instead of a probability. An odds is defined as the ratio of the probability of a positive outcome to that of a negative outcome. Therefore, the OR of 2.15 should be interpreted as “the *odds* of prostate cancer for hair dye users was *2.15-fold of* that for non-users”.

The epidemiological studies investigating the risk of cancer of hair dye use were mostly conducted in western countries. Because darker hair dyes that contain more aromatic amines are more often used in Asians than in Caucasians, the risk of cancer associated with hair dye use may be greater in Asians than in Caucasians. Nevertheless, caution should be exerted in applying Tai et al.*’s* study results until further evidence is available.

## Response to “Does hair dye use really increase the risk of prostate cancer?”

Shu-Yu Tai^2–6^, Hui-Min Hsieh^6, 7^, Shu-Pin Huang^8, 9^, Ming-Tsang Wu^5–7, 10^

^2^Graduate Institute of Medicine, College of Medicine, Kaohsiung Medical University, Kaohsiung, Taiwan

^3^Department of Family Medicine, School of Medicine, College of Medicine, Kaohsiung Medical University, Kaohsiung, Taiwan

^4^Department of Family Medicine, Kaohsiung Municipal Ta-Tung Hospital, Kaohsiung, Taiwan

^5^Department of Family Medicine, Kaohsiung Medical University Hospital, Kaohsiung Medical University, Kaohsiung, Taiwan

^6^Research Center for Environmental Medicine, Kaohsiung Medical University, Kaohsiung, Taiwan

^7^Department of Public Health, Kaohsiung Medical University, Kaohsiung Medical University, Kaohsiung, Taiwan

^8^Department of Urology, Kaohsiung Medical University Hospital, Kaohsiung Medical University, Kaohsiung, Taiwan

^9^Department of Urology, Faculty of Medicine, College of Medicine, Kaohsiung Medical University, Kaohsiung, Taiwan

^10^Graduate Institute of Clinical Medicine, College of Medicine, Kaohsiung Medical University, Kaohsiung, Taiwan

Shu-Yu Tai: shuyutai@gmail.com

Hui-Min Hsieh: hsiehhm@kmu.edu.tw

Shu-Pin Huang: shpihu@yahoo.com.tw

Ming-Tsang Wu: 960021@ms.kmuh.org.tw

**Corresponding Authors:** 960021@ms.kmuh.org.tw (Dr. Ming-Tsang Wu)

Room 721, CS Building, 100 Shih-Chuan 1st Road, Kaohsiung, Taiwan;

TEL: 886-7-312-1101 ext. 2141 ext. 55;

FAX: 886-7-3221806

### Abstract

The comments of Dr. Bang-Ping Jiann on our paper “Hair dye use, regular exercise, and the risk and prognosis of prostate cancer: multicenter case–control and case-only studies” discuss some important methodological issues such as the explanation of odds ratio (OR), the matching controls, and our handling of missing data. In our response, we will first focus on areas of agreement, then areas of disagreement, and end with issues requiring clarification or further development.

### Areas of agreement

Regarding the association between hair dye and cancers, we agree that some controversies still need to be clarified despite the positive association between hair dye and bladder cancers reported by case-control or meta-analysis studies from different countries [6–8]. Based on these epidemiologic studies and still many unknown risk factors related to prostate cancer, we conducted this case-control and case-only study to explore the association between the environment factors and the prostate cancer.

Dr. Jiann questioned whether or not dose-response relationship in our paper was assessed by duration and frequency, rather than cumulative exposure dose. Data about hair dyes use were collected by questionnaire, which made it impossible to calculate different levels of carcinogenic chemicals from various brands of or ingredients in the hair dyes We addressed this limitation in the Discussion in this paper [5].

Another issue was the interpretation of OR. Because this is a case-control study design, we agree that the sentence in the results section “*the development of prostate cancer in hair dye users was 2.15-fold higher than that in nonusers”* should be interpreted as “the *odds* of prostate cancer for hair dye users was *2.15-fold of* that for non-users” [9].

### Areas of disagreement

Dr. Jiann presents three key concerns, on which we do not agree. Below are our responses,

First, regarding the estimated prevalence of hair dye use in the 381 cases recruited from another hospital in the northern Taiwan was 23.1% (88/381), which was close to 21.6% (64/296) in the controls. Our study included two parts, the case-control study part and case-only study part. For the case-control study, we recruited newly pathologic proved prostate cancer from two medical centers in southern Taiwan and matched them 1:1 to one healthy men (control) who received health check-ups in the Department of Preventive Medicine during the same month according to age (in 2-year bands), ethnicity, and hospital of origin. Compared to patients in the control group, the ones in the case group had a higher rate of reported use of hair dyes from the two medical centers in southern Taiwan. Dr. Jiann pointed out that the estimated prevalence of hair dye use among the 381 cases recruited from another hospital in the northern Taiwan is close to 21.6% (64/296) in the controls. We think it was not appropriate to compare the above two, because these patients were from different hospital and not matched with age, ethnicity, and particularly hospital of origin. It could be that there was a lower prevalence of hair dye use in the matched control group from the northern area than that from the southern area of Taiwan. Alternatively, in our paper, we compared the prevalence of hair dyes use in 98 excluded cancer patients and those 296 studied cancer patients from the southern area and found the prevalence to be similar (28.6% vs. 32.1%) [5].

Second, Dr. Jiann highlights the possibility of recall bias and greater possibility of missing data regarding hair dye use in the case group than that in the control group. To deal with the missing data, we have presented another complete case analysis as a comparison, an analysis only including those subjects for which no data are missing, as suggested a reviewer. This was presented as supplementary material, and the results are similar with the original analysis (Supplemental Table 5 in [5]). Because the fraction of missing data in this study ranged between 0.08 and 5%, we also used the multiple imputation method to assign the missing values to the appropriate exposure variables [10]. Multiple imputation with five imputations for those samples are performed following standard rules described in a study by Rubin to achieve 98 to 99% relative efficiency to ensure in-range values [10]. We found that the results remain similar (Table 1), though the magnitudes of ORs are slightly lower than the original ones in Table 2 of our paper [5].

**Table 1:** Odds ratio (OR) for cases and controls according to hair dyes use and regular exercise after multiple imputation

Variables	Cases *N* = 296		Controls *N* = 296		Crude OR (95% CI)	AOR (95% CI)^a^	AOR (95% CI)^b^
	N	(%)	N	(%)			
Hair dyes							
No	201	(67.91)	231	(78.04)	1	1	1
Yes	95	(32.09)	65	(21.96)	1.62 (1.12–2.34)*	1.75 (1.16–2.63)**	1.93 (1.17–3.17)*
Age of first use (yrs)							
Never	207	(69.93)	234	(79.05)	1	1	1
≥ 60	27	(9.12)	22	(7.43)	1.39 (0.77–2.51)	1.18 (0.62–2.25)	1.35 (0.64–2.86)
50 - < 60	30	(10.14)	23	(7.77)	1.47 (0.83–2.62)	2.14 (1.07–4.27)*	2.31 (1.03–5.18)*
< 50	32	(10.81)	17	(5.74)	2.12 (1.14–3.94)	2.24 (1.17–4.26)*	1.98 (0.91–4.32)
P for trend					< 0.01	< 0.01	< 0.05
Years of use (years)							
Never	220	(74.32)	236	(79.73)	1	1	1
≤ 10	39	(13.18)	38	(12.84)	1.10 (0.68–1.78)	1.23 (0.72–2.11)	1.29 (0.68–2.43)
> 10	37	(12.50)	22	(7.43)	1.80 (1.03–3.15)*	1.90 (1.04–3.49)*	2.13 (1.03–4.41)*
P for trend					0.05	< 0.05	< 0.05
Frequency of use (times per year)							
Never	221	(74.66)	237	(80.07)	1	1	1
≤ 6	40	(13.51)	33	(11.15)	1.30 (0.79–2.13)	1.42 (0.83–2.44)	1.41 (0.75–2.65)
> 6	35	(11.82)	26	(8.78)	1.44 (0.84–2.48)	1.79 (0.98–3.31)	2.20 (1.05–4.62)*
P for trend					0.12	< 0.05	< 0.05
Year of first use							
No	216	(72.97)	234	(79.05)	1	1	1
After 1980	4	(1.35)	3	(1.01)	1.10 (0.67–1.78)	1.23 (0.72–2.11)	1.29 (0.67–2.43)
Before 1980	76	(25.68)	59	(19.93)	1.80 (1.03–3.15)*	1.90 (1.04–3.48)*	2.17 (1.03–4.41)*
P for trend					0.09	< 0.05	< 0.05

*Abbreviation*: *AOR* adjusted OR, *OR* odds ratio, *PC* prostate cancer

^a^Adjusting for age, and family history of PC, ^b^Adjusting for age, marital status, blood type, education, family history of PC, cigarette smoking, alcohol drinking and betel nut chewing

*P-value <0.05 **p-value < 0.01 ***p-value < 0.001

Third, Dr. Jiann also raised a concern about the small number of the hair dye users starting after 1980, and it may be inappropriate to compare the risk before and after the year of 1980. The manufactures complied with the U.S. Food and Drug Administration to reformulate the hair dye products to eliminate some of the ingredients that were carcinogenic during 1978 to 1982. We tried to provide more information about the association between the characters of hair dye use and prostate cancer regarding the important news announced by U.S. FDA. Our research findings should focus and interpret on the results before 1980, instead of the results after 1980 with few participants using hair dye and the wide 95% confidence interval [5].

### Areas requiring clarification or further development

A case-control study is an easier and less expensive way to help determine if an exposure is associated with an outcome than randomized control studies. Nevertheless, the case-control design may suggest association not prove the causation. Recall bias and lack of detail information of the exposure factors can be a particular concern in case-control study design. Therefore, further prospective cohort studies are necessary to confirm our findings.

## Abbreviations

CI: Confidence interval
OR: Odds ratio
